# Quality of Life in Patients With Unstable Angina Before and After Percutaneous Coronary Intervention: A Single-Center Pilot Study Using the European Quality of Life 5-Dimension 5-Level (EQ-5D-5L) Questionnaire

**DOI:** 10.7759/cureus.45886

**Published:** 2023-09-25

**Authors:** Bui Thanh Tin, Ho Tat Bang, Tran Thi Anh Thu, Nguyen Thi My Anh, Vu Hoang Vu, Nguyen Van Tap

**Affiliations:** 1 Preventive Medicine Department, Faculty of Public Health, University of Medicine and Pharmacy at Ho Chi Minh City, Ho Chi Minh City, VNM; 2 Thoracic and Vascular Department, University Medical Center Ho Chi Minh City, University of Medicine and Pharmacy at Ho Chi Minh City, Ho Chi Minh City, VNM; 3 Health Organization and Management Department, Faculty of Public Health, University of Medicine and Pharmacy at Ho Chi Minh City, Ho Chi Minh City, VNM; 4 Faculty of Public Health, University of Medicine and Pharmacy at Ho Chi Minh City, Ho Chi Minh City, VNM; 5 Health Communication Department, Medical Center of Binh Thanh District, Ho Chi Minh City, VNM; 6 Faculty of Nursing - Medical Technology, University of Medicine and Pharmacy at Ho Chi Minh City, Ho Chi Minh City, VNM; 7 Interventional Cardiology Department, University Medical Center Ho Chi Minh City, University of Medicine and Pharmacy at Ho Chi Minh City, Ho Chi Minh City, VNM; 8 Internal Medicine Department, Faculty of Medicine, University of Medicine and Pharmacy at Ho Chi Minh City, Ho Chi Minh City, VNM; 9 Faculty of Medical Management, Nguyen Tat Thanh University, Ho Chi Minh City, VNM

**Keywords:** vietnam, eq-5d-5l, coronary intervention, unstable angina, quality of life

## Abstract

Background

Unstable angina (UA) has a negative impact on patients’ quality of life. Percutaneous coronary intervention (PCI) is a commonly recommended treatment that exhibits positive therapeutic effects and enhances quality of life. This study aimed to compare the alterations in quality of life and related factors before and after PCI in UA patients.

Methods

A longitudinal follow-up study was conducted on 48 patients with UA before and one month after undergoing PCI. The European Quality of Life (EuroQol) 5-Dimension 5-Level (EQ-5D-5L) scale was utilized to measure the quality of life of patients.

Results

The study revealed a significant improvement in the quality of life score after one month of coronary artery intervention compared to the pre-intervention stage: the quality of life score before the intervention was 0.73 ± 0.32, whereas it increased to 0.89 ± 0.20 after one month (p<0.001). Sex, occupation, and troponin T were associated with changes in quality of life one month after the coronary artery intervention.

Conclusion

The pilot study demonstrated a notable enhancement in the quality of life among patients with UA following coronary intervention. Additionally, sex, occupation, and troponin T were identified as factors associated with this improvement.

## Introduction

Unstable angina (UA), non-ST-segment elevation (NSTEMI), and ST-segment elevation (STEMI) are all included in acute coronary syndrome. UA shows lower early mortality, but the long-term prognosis for these conditions is worse than for patients with ST-elevation myocardial infarction. The patients tend to be older, have more extensive coronary artery disease, and have more co-morbidities [1-3]. Patients with UA have a higher frequency of chest pain, dyspnea, and a lower quality of life than those with NSTEMI [3].

Percutaneous coronary intervention (PCI) is the most frequently indicated treatment for patients with UA worldwide [4]. It is one of the most effective treatments for coronary artery disease in the battle against coronary heart disease. The revascularization treatment strategy is associated with an improved quality of life and reduced symptom burden in these patients. Studies have demonstrated that PCI significantly improves the quality of life after six months and one year of follow-up [5,6]. However, there is little up-to-date evidence on changes in quality of life after treatment for patients with UA.

University Medical Center Ho Chi Minh City, a tertiary referral hospital in southern Vietnam, receives interventions for patients with coronary artery diseases. However, no in-hospital studies have evaluated the improvement in quality of life from coronary intervention for patients with UA. Therefore, this study evaluated the change in quality of life and related factors in patients with UA before and after coronary intervention.

## Materials and methods

Study settings and design

A longitudinal follow-up study was conducted on patients with UA at the Department of Interventional Cardiology, University Medical Center Ho Chi Minh City, Vietnam, from March to May 2023. The Ethics Council in Biomedical Research of the University of Medicine and Pharmacy at Ho Chi Minh City has approved this work (Decision number: 33/HĐĐĐ - ĐHYD signed on January 12, 2023). All procedures were performed according to The Declaration of Helsinki.

Participants, sample size, and sampling

We included inpatients ≥20 years of age in the Interventional Cardiology Department with a diagnosis of UA and who were indicated for coronary intervention. Before receiving a coronary intervention, we explained the purpose of the study, its benefits and risks, as well as the confidentiality of patient information. Additionally, we conducted a second interview with the patients one month after undergoing coronary intervention. Patients who died after the intervention or who did not participate in the post-intervention interview were excluded from the study.

The study used the formula to calculate the sample size for a paired samples t-test, with 𝜇_diff = 0.064 and 𝜎_diff = 0.15, based on Fearon et al.'s study [7]; Z(1-α/2) = 1.96, Z(1-𝛽) = 0.84. With a loss to follow-up rate of 10%, the minimum required sample size is 46 patients. The study sample conveniently selected all patients who met the sampling criteria during the study period.

Data collection and tool

Quality of life was assessed using The European Quality of Life 5-Dimensions 5-Level (EQ-5D-5L) instrument, which essentially consists of two domains: the EQ-5D descriptive system and the EQ visual analog scale (EQ VAS). The EQ-5D descriptive system consists of five dimensions: mobility, self-care, usual activities, pain/discomfort, and anxiety/depression. Each dimension has five levels: no problems, slight problems, moderate problems, severe problems, and extreme problems. The patient is asked to indicate their health state by ticking the box next to the most appropriate statement in each of the five dimensions. The EQ-VAS is a vertical visual analog scale that takes values between 100 (best imaginable health) and 0 (worst imaginable health) [7].

The variables we investigated include demographic characteristics such as age, group, sex, body mass index, educational level, residence, occupation, economic status, and marital status. The comorbidities and lifestyle habits (smoking, alcohol consumption, and exercise) were also investigated. The clinical and subclinical characteristics, including the number of narrowed branches, the number of revascularized coronary vessels, residual stenosis, and troponin T quantification, were collected. 

Statistical analysis

The research data were entered using the Epidata 4.6 software (EpiData Association, Odense, Denmark) and analyzed using the Stata 17.0 software (StataCorp, LLC, College Station, Texas, United States). Paired t-tests, ANOVA, and simple linear regression were employed to measure associations with a significance level of p < 0.05 when the quality of life scores were distributed normally. We use the Wilcoxon signed-rank test, the Kruskal-Wallis test, and the Spearman correlation to analyze the quality of life scores exhibited a non-parametric distribution.

## Results

Forty-eight patients with UA who met the selection criteria were enrolled during the study. Table 1 shows the demographic characteristics of the patients. The patients' mean age was 62.88 ± 11.33, with the youngest being 38 years old and the oldest being 83 years old. The majority of the participants were male (68.8%), and individuals aged 65 and above accounted for 41.7% of the group. The average BMI was 22.93 ± 3.20, and 50% of the patients were overweight or obese. Roughly 50% of the patients had completed high school, and 75% lived in different provinces or cities. More than half of the patients were retired, and the remaining were employed in various occupations, including farmers (8.3%), government employees (16.7%), self-employed workers (18.8%), homemakers (2.1%), and unemployed (2.1%). About 27.2% of the patients reported financial hardship, while the rest had a moderate economic status. Most patients were married, with only a small percentage having experienced divorce or being widowed (6.2%). The findings revealed that 35.4% of patients reported being current smokers, while among non-smokers, 16.7% had a history of smoking but had successfully quit. Moreover, 29.2% of the patients reported recent alcohol consumption within the last month. Interestingly, the proportion of patients engaging in daily physical exercise was 52.1%.

**Table 1:** Demographic characteristics (n=48).

Variable	N (%)
Sex	
Male	33 (68.8)
Female	15 (31.2)
Age group	
<55 years	10 (20.8)
55-64 years	18 (37.5)
≥65 years	20 (41.7)
Body mass index	22.93 ± 3.20
Educational level	
Under secondary school	15 (31.2)
Secondary school	9 (18.8)
High school or higher	24 (50.0)
Residence	
Ho Chi Minh City	12 (25.0)
Other provinces/cities	36 (75.0)
Occupation	
Farmer	4 (8.3)
Worker and employee	8 (16.7)
Self-employed	9 (18.8)
Homemaker	1 (2.1)
Retired/elderly	25 (52.1)
Unemployed	1 (2.1)
Economic status	
Poor	13 (27.1)
Sufficient	35 (72.9)
Marital status	
Single/divorced/widowed	3 (6.2)
Married	45 (93.8)
Smoke	
Yes	17 (35.4)
No	31 (64.6)
Drink alcohol	
Yes	14 (29.2)
No	34 (70.8)
Do exercise regularly	
Yes	25 (52.1)
No	23 (47.9)

Many patients with UA experience common conditions such as hypertension (77.1%) and dyslipidemia (68.8%). Diabetes mellitus (27.1%) is also prevalent among these patients. Additionally, chronic pulmonary disease (2.1%), chronic liver disease (4.2%), and chronic kidney disease (16.7%) are often associated with UA. Reports show that 22.9% of patients have a history of myocardial infarction, and 29.2% have undergone PCI (Table 2). 

**Table 2:** Comorbidities (n=48). PCI: percutaneous coronary intervention.

Variable	N (%)
Hypertension	37 (77.1)
Dyslipidemia	33 (68.8)
Diabetes	13 (27.1)
COPD	1 (2.1)
Chronic liver disease	2 (4.2)
Chronic kidney disease	8 (16.7)
Old myocardial infarction	11 (22.9)
Previously undergone PCI	14 (29.2)

Based on our analysis, the average troponin T level was 37.74 ng/L, with the lowest recorded at 5.04 ng/L and the highest at 225 ng/L. The majority of patients were diagnosed with triple-vessel disease, accounting for 47.9% of cases, while 29.2% had double-vessel disease, and 22.9% had single-vessel disease. After interventions, almost half of the patients underwent single-vessel intervention, while 31.3% underwent double-vessel intervention, 20.8% received triple-vessel intervention, and 72.9% of patients had residual stenosis (Table 3).

**Table 3:** Clinical, subclinical features, treatment (n=48).

Variable	N (%)
Troponin T	37.74 ± 52.65
Number of narrowed branches	
One branch	11 (22.9)
Two branches	14 (29.2)
Three branches	23 (47.9)
Number of revascularized coronary vessels	
One vessel	23 (47.9)
Two vessels	15 (31.3)
Three vessels	10 (20.8)
Residual stenosis	
Present	35 (72.9)
Absent	13 (27.1)

The study found that patients' quality of life scores increased significantly after undergoing PCI, from 0.73 ± 0.32 before treatment to 0.89 ± 0.20 after treatment (p<0.001), which indicates that PCI can improve patients' quality of life one month after the procedure. Additionally, the visual analog scale (VAS) score also showed a significant improvement (p<0.001), with the VAS score increasing from 65.94 ± 17.34 before the intervention to 81.77 ± 15.14 after the intervention (Table 4).

**Table 4:** EQ-5D and EQ-VAS Summary Index (n=48). *Wilcoxon signed-rank test.

Variable	Pre-intervention	Post-intervention	p-value*
EQ – 5D	0.73 ± 0.32	0.89 ± 0.20	<0.001
EQ-VAS	65.94 ± 17.34	81.77 ± 15.14	<0.001

In the domains of mobility, self-care, and usual activities, the proportion of patients experiencing issues before and after intervention is relatively low. However, concerning pain/discomfort (64.6%) and anxiety/depression (56.3%), problems before the intervention are quite high. Following the intervention, patients still encounter several challenges related to pain/discomfort (43.8%), while issues with anxiety/depression (18.8%) have significantly reduced (Table 5). 

**Table 5:** Health dimension of the EQ - 5D scale (n=48).

Variable	Pre-intervention n (%)	Post-intervention n (%)
Mobility		
No issue	31 (64.6)	37 (77.1)
Issue	17 (35.4)	11 (22.9)
Self-care		
No issue	36 (75.0)	43 (89.6)
Issue	12 (25.0)	5 (10.4)
Usual activities		
No issue	37 (77.1)	43 (89.6)
Issue	11 (22.9)	5 (10.4)
Pain/discomfort		
No issue	17 (35.4)	27 (56.2)
Issue	31 (64.6)	21 (43.8)
Anxiety/depression		
No issue	21 (43.8)	39 (81.2)
Issue	27 (56.2)	9 (18.8)

The research aimed to examine the factors that affected the quality of life of patients diagnosed with UA. The study analyzed how patients' quality of life scores differed before and after PCI. The results of the analysis are presented in Table 6. It was found that male patients experienced a greater improvement in their quality of life compared to female patients (p=0.025). This finding suggests that gender may influence the quality of life outcomes following PCI. Additionally, occupation was identified as another important factor that affected the improvement of patients' quality of life (p=0.046). Different occupational groups showed varying levels of improvement. The study also examined the relationship between troponin T levels and the improvement of quality of life after PCI. The analysis revealed a significant correlation coefficient of 0.362 and a p-value of 0.023, indicating that troponin T levels strongly influenced the extent of improvement in patients' quality of life. Patients with higher troponin T levels experienced a more significant improvement in their quality of life after the intervention.

**Table 6:** Factors associated with changes in quality of life score (n=48). *Wilcoxon signed-rank test; **Kruskal-Wallis test; ***Spearman rank correlation coefficient. IQR: interquartile range, Med: median.

Variable	Med (IQR)	p-value
Sex		
Male	0.09 (0 – 0.16)	0.025*
Female	0.15 (0.12 – 0.39)	
Occupation		
Farmer	0.04 (0 – 0.11)	0.046**
Worker and employee	0.06 (0 – 0.26)	
Self-employed	0.15 (0.12 – 0.16)	
Homemaker	0.29 (0.29 – 0.29)	
Retired/elderly	0.14 (0.07 – 0.27)	
Unemployed	0.18 (0.18 – 0.18)	
Troponin T	0.362***	0.023

## Discussion

The mean age of the study participants was 62.88 ± 11.33, consistent with several other studies on coronary heart disease [1,7-13]. Among them, the youngest age reported for the disease was 34, indicating a trend of younger patients with coronary heart disease. The study found a higher prevalence of the disease in males (68.8%) compared to females (31.2%), which aligns with results from most other studies on coronary heart disease [1,7,8,10,11,13]. Evidence from these studies suggests that males are more susceptible to coronary heart disease than females.

The proportion of smokers among the patients was 35.42%. However, the smoking rates varied in different studies due to differences in study design, variable definitions, duration, and geographical locations. For instance, Yan et al. [10] reported a smoking rate of 23.7%, Smedt et al. [9] reported 16.9%, and Fearon et al. [7] reported 19%, with a higher proportion of men. 

Among the participants, 6.3% had no comorbidities, while the rest had at least one comorbid condition. The most prevalent comorbidities in patients with UA were hypertension (77.1%) and lipid disorders (68.8%), while diabetes had an incidence rate of 27.1%. Comparing these findings with other studies, it is evident that hypertension rates consistently exceeded 50% in similar studies, while the rates of lipid disorders and diabetes may vary, but overall, these three conditions remain the most common comorbidities in patients with both UA and coronary artery disease [1,7,9-11,13]. These comorbidities contribute to the risk of coronary heart disease and significantly burden patients during treatment and healthcare management.

Among the study participants, 22.9% had undergone prior PCI. This rate was lower than found in studies in Vietnam [1,11], while studies conducted worldwide showed higher rates [7,10]. This difference can be attributed to factors such as race, geographical region, and study duration.

The research results demonstrated a statistically significant improvement in quality-of-life scores after one month of PCI intervention. The quality of life score before the intervention was 0.73 ± 0.32, which increased to 0.89 ± 0.20 after the intervention. These findings are consistent with a study by Jeong et al. [2], where the quality of life score before intervention was 0.77 ± 0.27 and 0.86 ± 0.21 after one month. The results indicate that PCI is an effective treatment method with positive outcomes and patient recovery.

Due to time limitations, our study could not assess the improvement in quality of life at similar timeframes as other studies, such as six months or one year after intervention. Improvements in quality of life are a long-term process involving physical, psychological, and health status factors, and long-term follow-up can provide a more accurate evaluation of the patient's quality of life. At 6 and 12 months, quality of life scores may differ in various studies. For instance, Fearon et al. [7] reported a decrease in quality of life score to 0.87 ± 0.15 after 12 months, while Azmi et al.'s study [14] on patients with acute coronary syndrome in Malaysia showed an increase from 0.75 at admission to 0.82 after 12 months. These variations could be due to differences in study location, duration, and the patient groups studied, as our study focused on patients with UA. In contrast, others examined patients with acute coronary syndrome or those without ST-segment elevation. Additionally, our study's relatively small sample size may have affected the obtained results. Furthermore, different versions of the EQ scale and various value conversion tables in different countries, based on their economic, cultural, and social characteristics, might explain differences in EQ scores among studies.

Factors related to changes in quality of life were found in the study, including sex, occupation, and troponin T. A study by Bakhai et al. [15] also revealed a correlation between sex and quality of life. Specifically, females had lower quality of life scores than males at 12 months after PCI, where women were generally older and had higher rates of hypertension and diabetes. Vu et al.'s study [11] also showed that females had poorer quality of life, especially 30 days after discharge, but they exhibited better physical recovery after 12 months compared to males, with no significant differences in other aspects. Van Nguyen et al.'s study [1] demonstrated a significant improvement in patients' quality of life with UA after three months of intervention, rather than one month, and found similar factors related to the quality of life as our study, such as troponin concentration. Occupation related to quality of life improvement is a novel finding in this research, not in other studies. Therefore, further studies are needed to evaluate the relationship between patients' occupations and their quality of life.

The EQ-5D-5L questionnaire reported that 50% of patients experienced pain/discomfort before intervention. However, our study only assessed the general level of pain/discomfort without a specific evaluation of chest pain due to coronary artery disease or other causes. Many studies have reported chest pain as a factor related to quality of life. The RITA-3 trial [6] indicated that each unit increase in the severity of chest pain was associated with a decrease of about five units in the EQ-VAS score (p<0.001) and a decrease of 0.068 points on the EQ-5D scale (p<0.001). According to this study, early intervention leads to greater improvements in quality of life compared to conservative treatment, and the quality of life improvement seems to be due to improvements in the level of chest pain. Spertus et al.'s study [13] concluded that the frequency of chest pain before surgery was the most important prognostic factor for improved quality of life after PCI.

This study offers important insights into the impact of coronary artery intervention on the quality of life of patients with UA. The findings are practical for hospitals, as they can inform specialists' understanding of patients' conditions and the effectiveness of treatment. By making appropriate recommendations and solutions, specialists can improve the quality of life for these patients, increasing their life expectancy and bringing them closer to optimal health. One limitation of this study is its short duration and the small sample size, which may not accurately represent the target population. Additionally, the EQ-5D-5L tool only assesses the patient's quality of life in five areas, including pain and discomfort. It does not specifically evaluate the patient's chest pain, which could be assessed using other specialized scales.

## Conclusions

Patients with UA experience a decrease in their quality of life. The study demonstrated a notable enhancement in the quality of life among patients with unstable angina following coronary intervention. Additionally, sex, occupation, and troponin T were identified as factors associated with this improvement.
